# Lineage-specific SNPs for genotyping of *Mycobacterium tuberculosis* clinical isolates

**DOI:** 10.1038/s41598-017-01580-z

**Published:** 2017-05-03

**Authors:** Horng-Yunn Dou, Chien-Hsing Lin, Yih-Yuan Chen, Shiu-Ju Yang, Jia-Ru Chang, Keh-Ming Wu, Ying-Tsong Chen, Pei-Ju Chin, Yen-Ming Liu, Ih-Jen Su, Shih-Feng Tsai

**Affiliations:** 1 Institute of Molecular and Genomic Medicine, National Health Research Institutes, Zhunan, Miaoli Taiwan; 2 Genome Research Center, National Yang-Ming University, Taipei, Taiwan; 3 Institute of Bioinformatics, National Chung Hsing University, Taichung, Taiwan; 4 Institute of Infectious Diseases and Vaccinology, National Health Research Institutes, Zhunan, Miaoli Taiwan; 5 Institute of Biomedical Informatics, National Yang-Ming University, Taipei, Taiwan; 6 Department of Biochemical Science and Technology, National Chiayi University, Chia-Yi, Taiwan

## Abstract

Tuberculosis (TB) is a severe infectious disease worldwide. Genetic variation of the causative agent, *Mycobacterium tuberculosis* (MTB), determines the outcomes of infection and anti-TB treatment. Until recently, there has been no effective and convenient way for classifying clinical isolates based on the DNA sequences of the divergent lineages of MTB infecting human populations. Here, we identified single nucleotide polymorphisms (SNPs) of six representative strains from Taiwan by whole-genome sequencing and comparing the results to the sequence of the H37Rv reference strain. One hundred and ten SNPs, each unique to one of the six strains, were used to genotype 150 additional isolates by applying DNA mass spectrometry. Lineage-specific SNPs were identified that could distinguish the major lineages of the clinical isolates. A subset including 32 SNPs was found to be sufficient to type four major groups of MTB isolates in Taiwan (ancient Beijing, modern Beijing, East African–Indian, and Latin-American Mediterranean). However, there was high genetic homozygosity within the Euro-American lineage, which included spoligotype-classified Haarlem and T strains. By whole-genome sequencing of 12 representative Euro-American isolates, we identified multiple subtype-specific SNPs which allowed us to distinguish two major branches within the Euro-American lineage.

## Introduction

Tuberculosis (TB) is a worldwide health-care concern. It has been characterized by the World Health Organization (WHO) as an epidemic, with an estimated one-third of the world’s population having been infected by the causative agent *Mycobacterium tuberculosis* (MTB)^1^. Epidemiologic studies have revealed that various genotypes of MTB may be prevalent in different geographic regions and that genotype distribution can be associated with population migrations^2–4^. The extent to which MTB genomic diversity influences human disease in clinical settings remains an open question.

The development of molecular techniques for differentiating various MTB isolates is of considerable interest in epidemiological studies. Genotyping methods aimed at generating phylogenetically informative data have been developed to investigate multiple MTB clinical samples from different sources. In past decades, the restriction fragment length polymorphism (RFLP) method based on IS6110 was commonly used to study TB transmission by tracking individual strains of MTB in the community^5^. Currently, two genotyping methods are commonly applied to study MTB transmission^6^. Spoligotyping is based on polymorphisms in the direct repeat (DR) locus, which consists of 36-bp DR copies interposed by non-repetitive spacer sequence. It is a PCR-based reverse hybridization technique. The portable data format facilities easy inter-laboratory comparison. Freely accessible databases for strain lineage identification have been developed that involve spoligotype signature matching^7^. A second molecular technique for MTB strain typing is based on variable number tandem repeats (VNTRs) of mycobacterial interspersed repetitive units (MIRUs)^8–10^. This method determines the number of repeats at each of 12, 15 or 24 selected MIRU loci, also by using PCR.

We previously demonstrated that the Beijing ancient strain and the Haarlem strain are the predominant MTB strains infecting aborigines in eastern Taiwan (Hualien City), whereas the East-African Indian (EAI) strain is prevalent in southern Taiwan aborigines, and the Beijing modern strain is predominant in Han Chinese^11–14^. Taiwan is a relatively isolated island, serving as a mixing vessel for colonization by different waves of ethnic and migratory groups over the past 4 centuries. By using molecular methods, we previously identified close associations of the major MTB lineages in Taiwan (Beijing, EAI, Haarlem, and Latin-American Mediterranean (LAM) strains) to historical migrations of different ethnic populations to the island^14^. In the present study, six MTB strains – isolates of the Beijing ancient sublineage, the Beijing modern sublineage, Haarlem, EAI, T1, and LAM – representing the major types of clinical strains isolated from three different ethnic groups (aboriginals, Han Chinese, ‘veterans’) in Taiwan^11, 12^ were subjected to whole-genome sequencing using next-generation sequencing technology (Roche 454/Illumina GAIIx)^15–17^. Based on comparative genome analysis, we identified 60 and 141 strain-specific single nucleotide polymorphisms (SNPs) in the PE/PPE and non-PE/PPE gene families, respectively, when we compared these six genomes to that of the H37Rv reference strain. We then used 110 lineage-specific SNPs as markers to design a novel strain classification scheme and conduct phylogenetic analyses. The performance of this genotyping panel was compared with the results of spoligotyping on 156 MTB complex (MTBC) isolates.

## Results

### Genome sequencing of six MTB clinical isolates

**Figure 1:**
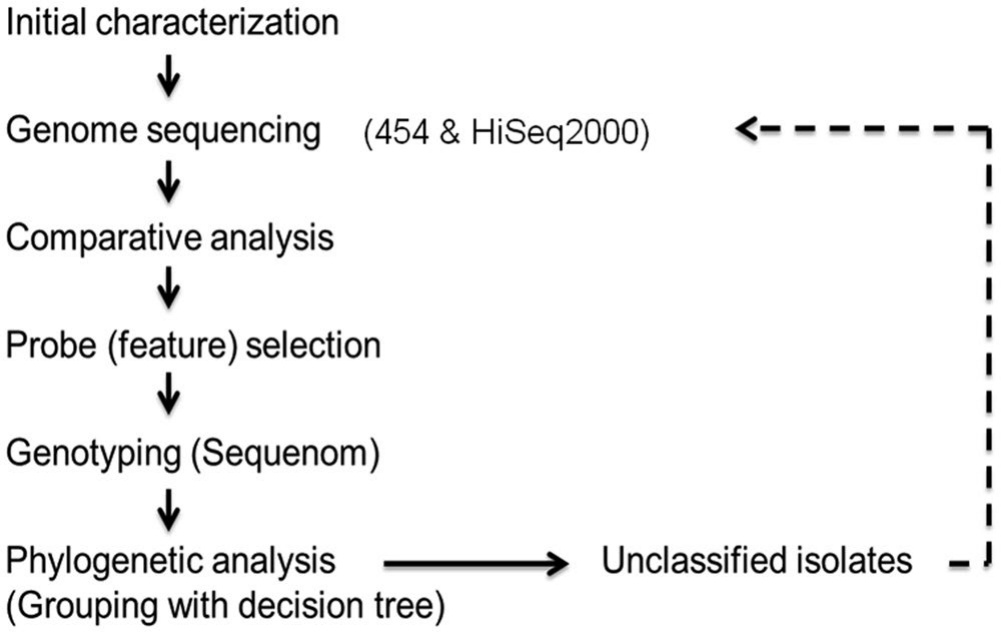
Overall scheme for selecting lineage-specific DNA markers. Initial characterization of the MTB strains involved spoligotyping and MIRU typing, but ethnic background of the infected patients was also considered^13–16^. Based on those data, we initially selected six MTB strains for whole-genome sequencing using 454 pyrosequencing technology. The genome sequence of each strain was then compared to that of the reference genome H37Rv to identify lineage-specific SNPs. Primers were designed to identify 110 SNPs (and a 25-tagSNP), which were then used for genotyping of 156 MTB isolates by MassARRAY mass spectrometry (Sequenom). Phylogenetic trees were generated based on the SNP genotypes. Some strains were not readily classifiable, so the genomes of representative isolates were sequenced (by using a HiSeq 2000 platform) to identify SNPs for these strains, and the phylogenetic analysis was repeated on them.

The overall scheme we used for selecting lineage-specific DNA markers is shown in Fig. 1. Based on our previous studies of MTB stains in Taiwan^13–16^, we selected six representative strains for whole-genome sequencing. The initial classification of these bacteria was based on spoligotyping and MIRU-typing, but we also considered the ethnic background of the patients infected with MTB. We applied a whole-genome shotgun approach to generate high-coverage sequences using 454 pyrosequencing technology^18^. Three of the isolates (W6, M3, M7) were sequenced by using a 454 GS20 sequencer, with an average read length of 96 bp. The other three isolates (A27, A18, M24) were sequenced by a 454 FLX sequencer, with a longer average read length of 227 bp and fewer sequencing runs. The sequencing depths were about 14~23-fold in the 454 GS20 data and about 16~28-fold in the 454 FLX data. The mapping results are summarized in Supplementary Table 1.

For each of the six isolates, the mapping rate was at least 95.8% and covered ≥97% of the H37Rv reference sequence (4.41 Mb). The total numbers of contigs for the three isolates sequenced by 454 GS20 were 214~305, and for the three isolates sequenced by 454 FLX, they were 290~299. The numbers of large contigs (≥1,000 bp) were 134~158 for the three isolates sequenced by 454 GS20 and 196~200 for the three isolates sequenced by 454 FLX, indicating that the proportion of large contigs was greater for the 454 FLX sequencer. The base quality evaluations for the large contigs, as measured by Phred scores of ≥40 (Q40Bases), were 99.48% to 99.95% in the six isolates, indicating that the sequence quality is high.

### Genetic variation in the MTB clinical isolates

We next compared the genome sequence of each of the six strains to the H37Rv reference genome to detect variant sequences for each isolate. We extracted a total of 9,003 high-confidence (HC) variations (for the definition of HC variants, see the Methods section), including SNPs, multiple nucleotide polymorphisms (MNPs), insertions and deletions (INDELs), from the mapping results of the six isolates. After sorting these variations according to the reference positions, and requiring that at least one isolate have >80% variation frequency at the position, we were left with 3,819 reference positions for which all six isolates had at least three reads. For simplicity, we chose 3,582 reference positions represented by SNPs for the following analyses (of the remaining positions, 27 were INDELs and 210 were MNPs).

Among these 3,582 SNPs, 404 are shared in all six isolates, and 13, 19, 232, 538 SNPs exist in five, four, three, and two of the six isolates, respectively (details are shown in Supplementary Table 2). The most abundant SNPs are 2,376 strain-specific polymorphisms (i.e., HC differences exist only in one of the six strains) and we used them as candidates for seeking lineage-specific SNPs. We divided these candidate SNPs into three main categories according to their locations in coding or non-coding regions: PE/PPE gene family, non-PE/PPE gene category, and intergenic SNPs (as shown in Table 1). For SNPs in coding regions, in general, non-synonymous SNPs are more prevalent than synonymous SNPs. The PE/PPE gene family comprises about 10% of the coding capacity of the MTB genome^19^; thus, to a first approximation, there are proportionately fewer SNPs in the PE/PPE family than in the non-PE/PPE gene category.

**Table 1:** Statistics of lineage-specific single nucleotide polymorphisms (SNPs).

Isolate	Lineage	No. of lineage-specific SNPs	PE/PPE gene family		non-PE/PPE gene family		
			Synonymous SNPs	Non-synonymous SNPs	Synonymous SNPs	Non-synonymous SNPs	Intergenic SNPs
M3	*Haarlem*	133	3	3	55	56	16
W6	*modern Beijing*	270	4	7	78	150	31
M7	*LAM*	317	10	7	93	163	44
A18	*EAI*	1,260	37	60	368	639	156
A27	*T*	136	2	2	48	69	15
M24	*ancient Beijing*	260	6	10	78	138	28
Sum		2,376	62	89	720	1,215	290

### SNP genotyping of 156 MTB clinical isolates

**Figure 2:**
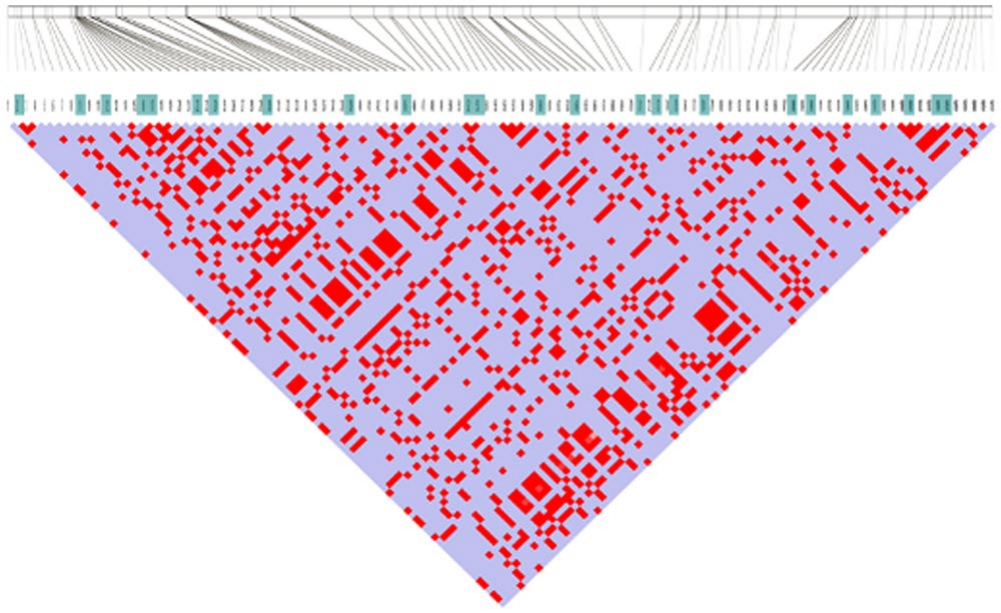
Linkage disequilibrium of SNP markers in the studied MTB genomes. The LD plot was created using Haploview software, and the color code on the plot follows the standard color scheme for Haploview: blue indicates |D′| = 1 and LOD < 2, and bright red indicates |D′| = 1 and LOD ≥ 2.

To characterize SNPs in 156 clinical isolates for phylogenetic analysis, we initially selected 120 lineage-specific SNPs with HC scores to design primers for Sequenom MassArray assays. These 120 lineage-specific SNPs were unequally selected from the six lineage isolates, as shown in Table 2, which was a reflection of the differences in the total numbers of lineage-specific SNPs between them. These 120 SNPs were divided into two categories^6^: 60 SNPs within the PE/PPE gene family (40 non-synonymous and 20 synonymous)^9^ and 60 of 1,215 non-synonymous SNPs from the non-PE/PPE gene category (details are shown in Supplementary Table 3). Five of the 120 SNPs were not appropriate for the Sequenom matrix-assisted laser desorption ionization–time of flight mass spectrometry (MALDI–TOF) system because of high GC content and/or primer dimer formation. The remaining 115 SNPs were designed to be detectable in 10 multiplex reactions, and were applied to genotype the 156 clinical MTB isolates. We excluded five of these SNPs because of low call rates (<95%) and bad clustering patterns, leaving 110 SNPs in the following analysis. The false-positive and false-negative rates were both 0% when comparing the Sequenom and 454 sequencing data, and the average call rate of each of the 110 SNPs in the 156 samples was 97%. There were strong correlations between these SNPs based on linkage disequilibrium analysis, as shown in Fig. 2. These 110 lineage-specific SNPs were completely tagged by 25 tagSNPs, with *r* ^2^ = 1.

**Table 2:** Selection of lineage-specific SNPs for strain typing.

Strain	M24	W6	A18	M7	M3	A27
Lineage	*ancient Beijing*	*modern Beijing*	*EAI*	*LAM*	*Haarlem*	*T*
No. of original lineage-specific SNPs	260	270	1,260	317	133	136
No. of designed SNPs	25	17	31	17	16	14
No. of actual genotyped SNPs	22	17	29	15	15	12
No. of SNPs with 100% variant frequency in other isolates	7	3	19	3	0	0

### Phylogenetic and grouping analysis of MTB isolates

**Figure 3:**
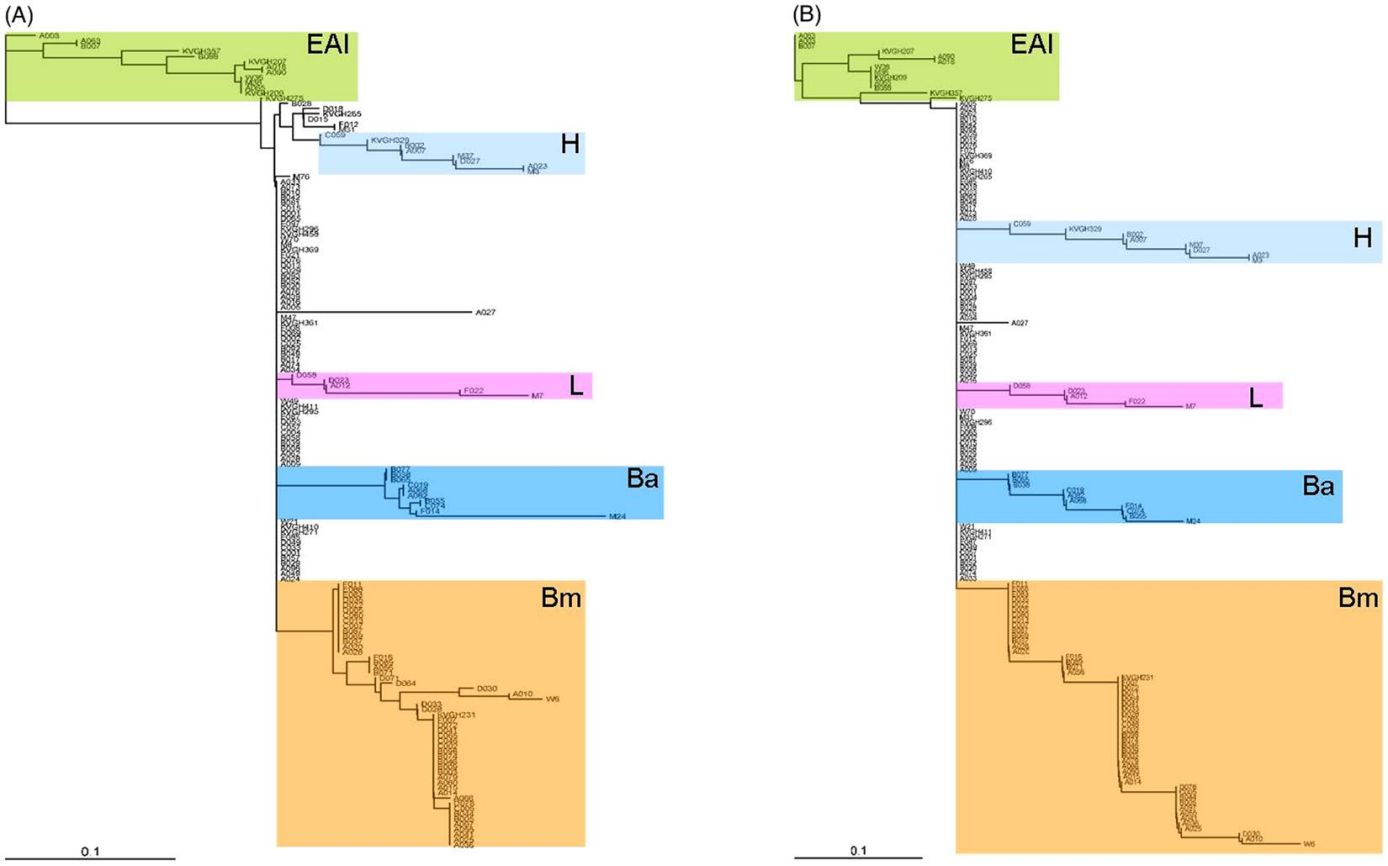
Phylogenetic analysis of MTB isolates using strain-specific SNP markers. PHYLIP software was applied to calculate the Nei’s distance using 110-SNP (**A**) and 25-tagSNP (**B**) data, and then phylogenetic trees were constructed using the neighbor-joining approach. Ba, ancient Beijing; Bm, modern Beijing; EAI, East African–Indian; H, Haarlem; L, Latin-American Mediterranean.

To trace the relationships between the 156 clinical isolates, we constructed phylogenetic trees based on 110-SNP or 25-tagSNP information, as shown in Fig. 3. Although the total numbers of markers used were different between these two trees, the morphology of the 25-tagSNP phylogenetic tree is the same as that of the 110-SNP tree, indicating that the 25 tagSNPs can accurately represent the genomic variations among strains. Based on preliminary lineage information from spoligotyping data for 107 of the clinical isolates, we assigned 75 of them (10 ancient Beijing, 51 modern Beijing, 11 EAI and 3 LAM) to the appropriate branches of both phylogenetic trees (Fig. 3). In addition, we were able to assign 6 spoligotype-unclassified isolates as belonging to the modern Beijing (n = 2), EAI (n = 2) and LAM (n = 2) lineages based on the nodes of the phylogenetic trees.

**Figure 4:**
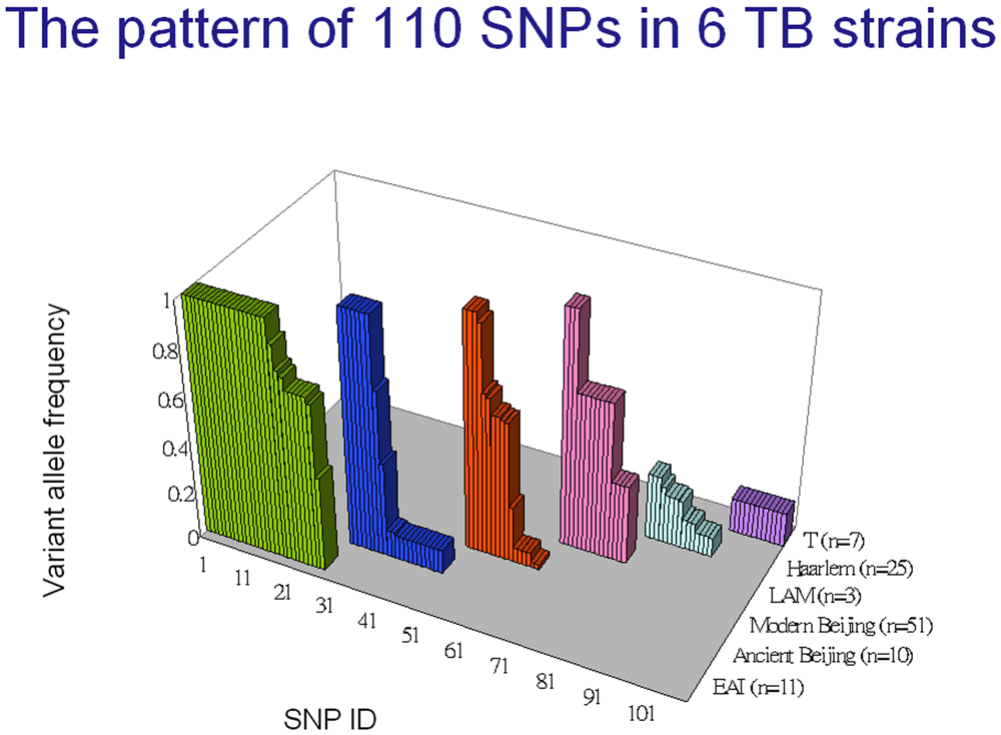
Identification of specific markers for strain typing. The allele frequencies of the 110 SNPs in 51 modern Beijing, 25 Haarlem, 11 EAI, 10 ancient Beijing, 7 T and 3 LAM isolates were characterized by combining spoligotyping and SNP genotyping data.

**Figure 5:**
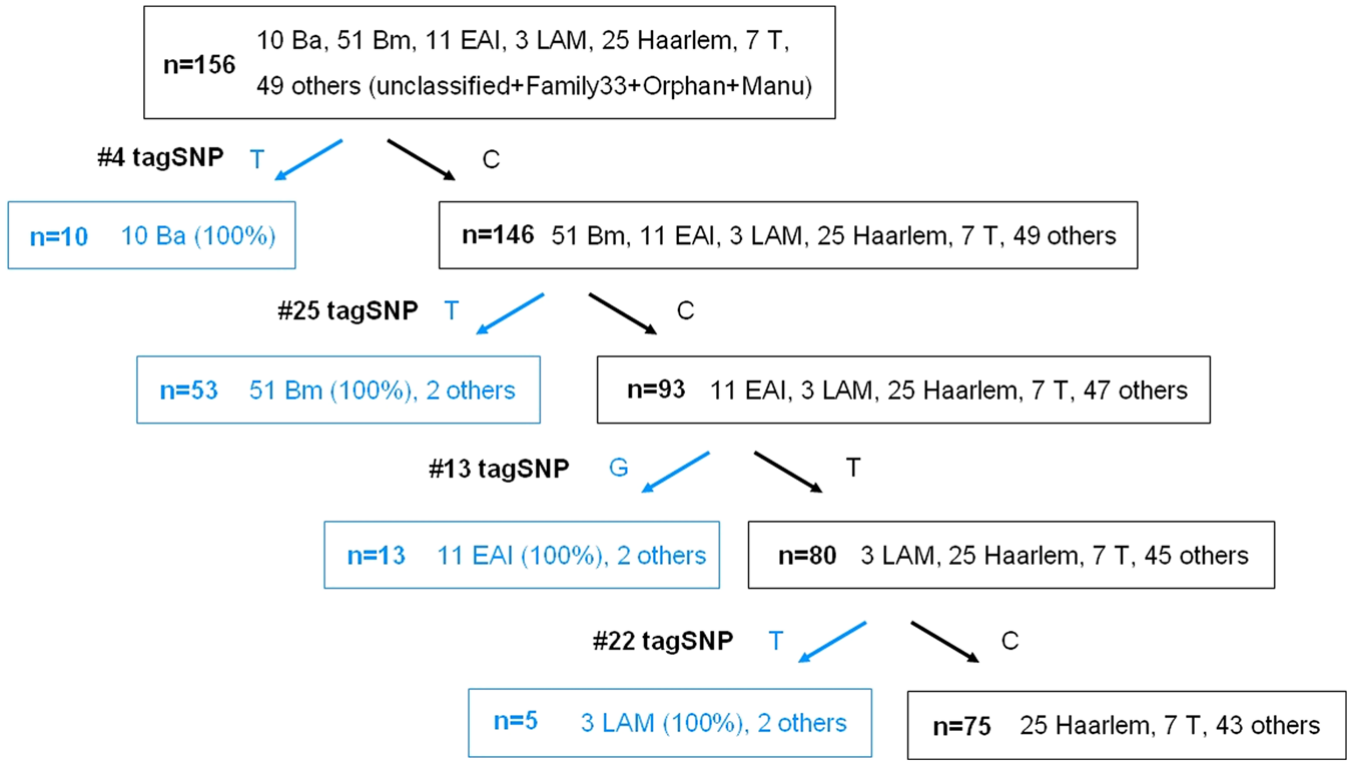
Decision tree based on four lineage-specific SNP markers. Four of 32 lineage-specific SNPs with 100% variant allele frequencies were used to classify 81 clinical isolates into ancient Beijing (Ba), modern Beijing (Bm), East African–Indian (EAI) and Latin American and Mediterranean (LAM) lineages.

By combining spoligotyping and SNP genotyping data, we characterized the allele frequencies of the 110 SNPs in 51 modern Beijing, 25 Haarlem, 11 EAI, 10 ancient Beijing, 7 T and 3 LAM isolates (i.e., the same 107 clinical isolates studied above). As shown in Fig. 4, all 110 SNPs were lineage-specific in these strains and showed polymorphisms in the corresponding lineage. Importantly, 32 SNPs were consensus variants in MTB lineages (Table 2): 7 SNPs were lineage-specific in ancient Beijing, 3 in modern Beijing, 19 in EAI, and 3 in LAM. Therefore, each of these 32 SNPs can be used to represent the MTB lineage of its corresponding strain (i.e., ancient Beijing, modern Beijing, EAI or LAM), and we constructed a decision tree based on four lineage-specific SNP markers (Fig. 5).

**Figure 6:**
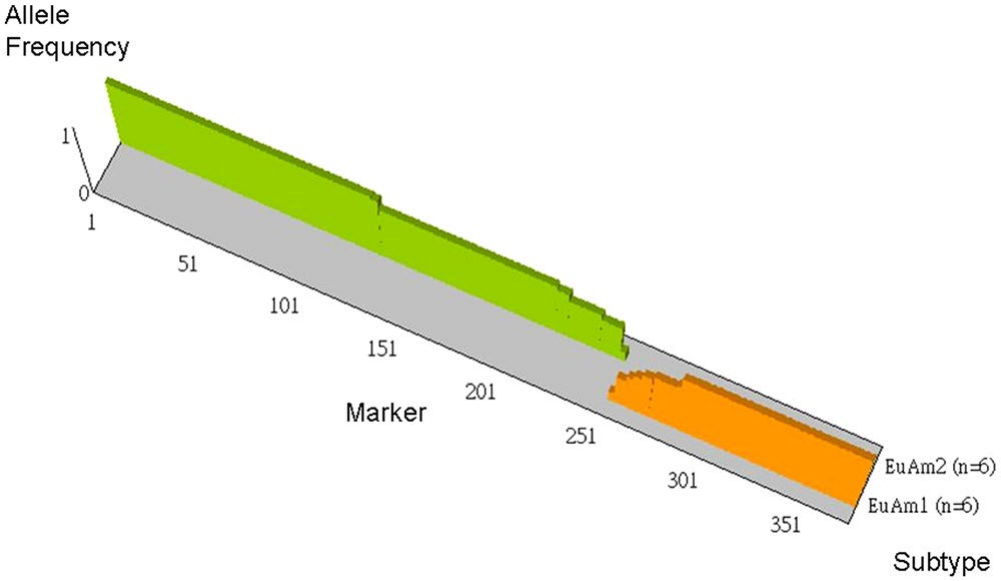
High genetic homozygosity within newly proposed Euro-American subtypes. The genomes of 14 Euro-American strains (7 Haarlem and 7 T) were sequenced using 454 or HiSeq2000 sequencing technology. Two major clusters (6 and 6 belong to the EuAm1 and EuAm2 subtypes, respectively) were identified based on the phylogenetic tree (Supplementary Figure 3). There were 81 EuAm1-specific and 133 EuAm2-specific SNPs with variant allele frequency = 100%.

Thirty-two of the 107 (30%) spoligotype-classified isolates were poorly classifiable using these 25-tagSNPs, and these isolates all belong to the Euro-American lineage (25 and 7 were classified as Haarlem and T strains, respectively, based on spoligotype data). We hypothesize that there are high homozygosities of spoligotype markers within Haarlem or T strains, resulting in the absence of leaf nodes for these strains on the decision tree (Fig. 5). To explore the genomic diversities of the Euro-American lineage, we applied whole-genome sequencing to characterize the genomic profiles of six Haarlem and six T strains. We identified 4,419 SNPs in these 12 Euro-American strains (Supplementary Table 4). We combined the SNP information of M3, A27 (454 sequencing data) and these 12 isolates (HiSeq2000 sequencing data) to construct a phylogenetic tree and perform principal component analysis, and found that several strains with the same spoligotype are not well clustered (Supplementary Figure 1). These results demonstrate that there are high homozygosities within Euro-American lineages, including Haarlem and T subtypes, a conclusion which is also supported by the 24-MIRU-VNTR phylogenetic tree (Supplementary Figure 2). Importantly, the M3 and A27 isolates, which were used to identify lineage-specific SNPs and construct the decision tree (Supplementary Figure 1), were clustered together, but some Haarlem and T isolates were distant from M3 and A27, accounting for the absence of a leaf in the decision tree for classifying these two subtypes. In addition, there were two major clusters comprising the Euro-American phylogenetic tree (Supplementary Figure 3), which we named the EuAm1 and EuAm2 subtypes. Based on this newly proposed definition of EuAm subtypes, there is high homozygosity within each EuAm subtype, as shown in Fig. 6, and only two SNPs are needed to classify Euro-American strains into these two hypothetical subtypes.

## Discussion

Tuberculosis remains a major public health issue in Taiwan and throughout the world^11, 12, 20^. Over the past years, the development of genotyping methods for molecular epidemiological study of TB has advanced our understanding of the transmission of MTB in human populations. Classification of strains into sub-lineages provides perspectives on the phenotypic consequences of genetic variations of the MTB strains. Phylogenetic analyses of MTB strains have also offered new insights regarding the evolution of MTB and the existence of distinct clades. From a public health perspective, an ideal methodology to determine the genetic variation of MTB clinical isolates should be simple, rapid, and affordable, and the results should be transferrable in a format that can be easily shared among laboratories. In this study, we designed a selection scheme for lineage-specific markers by applying whole-genome sequencing, comparative analysis, and genotyping with DNA mass spectrometry, and, furthermore, demonstrated the utility and accuracy of this new typing protocol. Because of its speed and ease of laboratory operation, and the simple data format for exchange and comparison, the protocol reported here has the potential to become a new standard method. It should also prove valuable for the development of an effective infection-control policy.

Although spoligotyping analysis is a straightforward technique, it is less discriminatory than IS6110 RFLP analysis. Moreover, it is a labor-intensive and time-consuming procedure. Even though strain classification based on spoligotyping can assign MTBC strains to the correct phylogenetic lineages in about 90% of cases, some strains cannot be classified at all^21^, and others might be misclassified, as we found in the present study (Supplementary Figure 2). Analysis of MIRU-VNTR loci is reproducible and sensitive, and it provides better resolution than spoligotyping. However, depending on the context, such investigations can be less than or as discriminatory as IS6110 RFLP. Strain-specific SNP typing can provide precise sequence-based information, and could be automated for large-scale studies of molecular epidemiology and phylogenetics. The combination of spoligotyping and MIRU-typing can be a cost-effective method for MTB genotyping. However, spoligotyping fails to sort about 20–40% of strains^12, 22, 23^, and there is no way to rectify this limitation. The MIRU–VNTR typing method does not sufficiently differentiate many Beijing genotype strains; therefore, it cannot be used for routine epidemiological study in areas where the Beijing genotype is prevalent. The addition of several VNTR loci is required to use VNTR typing as a routine epidemiological tool without doing RFLP analysis^20, 24^.

Additional genotyping of MTB isolates is essential for understanding the dynamics of transmission. Genetic information will help determine precise quantitative measures for transmission dynamics and augment classical epidemiological models. The ability to assess inter-strain genetic relationships provides a powerful means for resolving several key epidemiological issues such as: determining sources of infection; differentiating recent transmission from reactivation, or reinfection from relapse or treatment failure; tracing of chains of transmission; monitoring geographic distribution and spread of particular genetic strains; or investigating the evolution of MTB. For example, a fascinating study by Comas *et al*. compared MTBC phylogenetic diversity to human diversity inferred from mitochondrial genome data^25^. It will be interesting to investigate MTB phylogenic diversity in relationship to Taiwan ethnic diversity based on mitochondrial genome data.

The proposed workflow of selecting lineage-specific DNA markers (Fig. 1) is an effective and logical way to discriminate MTB isolates into genetic subtypes. Through the iteration of this workflow, we successfully found that only six markers are needed to classify clinical MTB isolates into six types, including ancient and modern Beijing, EAI, LAM, EuAm1 and EuAm2. Importantly, the concept of our workflow is also applicable in other fields of microbial research, e.g., searching highly conserved domains of variable clinical isolates in the context of vaccine development. Based on SNPs of isolated strains, several other research groups have devised new typing methods and applied them for classifying MTB strains^26, 27^. Notably, Coll *et al*. applied 92k SNP typing across a global collection of 1601 genomes and used 7 k strain-specific SNPs to discriminate known circulating strains^28^. A limitation of our study is that the collection of strains analyzed, although representative of the major MTB strains in Taiwan, is not representative of the global diversity of MTB.

## Methods

### Bacterial strains and molecular typing

MTB isolates were collected between 2004 and 2007 from the mycobacteriology laboratories of five general hospitals located in four geographical regions in Taiwan, namely, Taipei Tri-Service General Hospital (northern region), Mennonite Christian Hospital (eastern region), Wan-Ciao Veterans Hospital (central region), Tainan Chest Hospital (southern region), and Kaohsiung Veterans General Hospital (southern region). The bacterial strains used in this study are representative of the diversity of MTB in Taiwan as shown previously^11, 12, 20, 24^. Spoligotyping and MIRU-VNTR genotyping assays were performed based on internationally standardized protocols^6, 7^. In total, 156 isolates (of the Beijing, EAI, Haarlem, LAM, T, MANU, and unclassified strains) that had all genotype data available were used for the analyses. This study was approved by the Human Ethics Committee of the National Health Research Institutes, Taiwan (Code: EC0961103).

### Genome sequencing of MTB strains

Six MTB strains, W6, M3, M7, A27, A18 and M24, belong to the genogroups modern Beijing, Haarlem, Latin-American Mediterranean (LAM), T, East African-Indian (EAI), and ancient Beijing, respectively. They represent the major types of clinical strains isolated from three different ethnic groups in Taiwan and were subjected to whole-genome sequencing using the 454 pyro-sequencing approach^18^. The MTB genomes were sequenced to depths of 14- to 28-fold separately using a Genome Sequencer 20 (GS-20) or a Genome Sequencer FLX (GS-FLX) instrument (454 Life Sciences, Roche)^18^ with a 500–800 base-pair shotgun library for each strain.

DNA libraries of six Haarlem and six T clinical isolates were prepared using a Nextera DNA sample preparation kit (Illumina, San Diego, CA), and were multiplex-sequenced (2 × 100 bp) in one lane of a flow cell using a HiSeq2000 sequencer. After performing the de-multiplexing procedure, the average sequence size of each sample was 3.38 Gb, and the depths of these samples ranged from 568- to 1068-fold when mapped to the H37Rv reference sequence, resulting in a reference coverage of these samples of 99.44% to 99.82%. The detailed information was described in our recent article^29^.

### Mapping to the reference genome H37Rv

The 454 sequencing raw data (sff files) from each strain were collected into a specific folder as the read source to align to the H37Rv reference genome. The H37Rv genome sequence and the annotated gene information were downloaded from the NCBI ftp site for Microbial Genome Assembly/Annotation Projects (ftp://ftp.ncbi.nih.gov/genomes/Bacteria/Mycobacterium_tuberculosis_H37Rv_uid57777/). 454 GS Reference Mapper (Roche) software (version 2.3) was used to map the 454 reads to the reference sequence (see Table 1 for detailed information) and generate high-confidence variations between the reference and each of our six MTB clinical strains.

### Selection of strain-specific SNPs

The result file “454HCDiffs.txt” contains “High-Confidence” differences with at least three non-duplicate reads that (a) show the differences, (b) have at least five bases on both sides of the difference, (c) have few other isolated sequence differences in the read, and (d) have at least one aligned in the forward direction and at least one aligned in the reverse direction. In addition, only those variation sites for which all six strains have at least three reads covered and the variation rate is larger or equal to 80% were considered as valid. In-house scripts were used to merge the mapping results of all six strains and parse those valid differences into a MySQL database for further analysis. Strain-specific (observed only in a single strain) SNPs were selected and grouped into two categories: PE/PPE protein family and non-PE/PPE. According to the location of the variations, they can be synonymous or non-synonymous to the coding sequences. Furthermore, in the non-PE/PPE group, the variations can reside within non-coding sequences, which are intergenic regions. For further confirmation using MassARRAY Analyzer (Sequenom), the number of variations was reduced with the criteria that both total depth and variation depth must larger than 15, and the variation frequency must greater than 90% for each variation site.

For SNP calling of Illumina HiSeq2000 sequence data, mapped sequence data of each sample were analyzed using CLC Genomics Workbench software (Aarhus, Denmark) with default parameters. We applied an additional filter to identify highly reliable SNPs with more than 30-fold depth and >95% variant frequency.

### SNP genotyping based on the MassArray system

PCR and extension primers were designed for 60 PE/PPE and 60 randomly selected non-PE/PPE SNPs using MassArray Assay Design 3.1 software (Sequenom, San Diego, CA). Five were excluded due to difficult sequences. PCRs contained, in a volume of 5 µl, 1 pmol of the corresponding primers, 10 ng genomic DNA, and HotStar reaction Mix (Qiagen) in 384-well plates. PCR conditions were as follows: 94 °C for 15 min, followed by 40 cycles of 94 °C (20 s), 56 °C (30 s), 72 °C (60 s), and a final extension of 72 °C for 3 min. In the primer extension procedure, each sample was denatured at 94 °C, followed by 40 cycles of 94 °C (5 s), 52 °C (5 s), 72 °C (5 s). The mass spectrum from time-resolved spectra was retrieved by using a MassARRAY mass spectrometer (Sequenom), and each spectrum was then analyzed using SpectroTYPER software (Sequenom) to perform the genotype calling. After analyzing the genotype profiles, the clustering patterns of five SNPs could not be used to correctly perform genotype calling, and the data of 110 SNPs (57 PE/PPE and 53 non-PE/PPE) were finally used in the following analyses.

### Linkage disequilibrium and phylogenetic analysis

Based on Haploview software^30^, the Lewontin D’ measure was used to estimate the intermarker coefficient of linkage disequilibrium (LD) as shown in Fig. 2. An extra-stringent criterion, *r* ^2^ = 1 between each pair markers, was used to select 25 tagSNPs from 110 SNPs. We applied PHYLIP software to calculate the Nei’s distance using SNP data^31^, and then constructed a phylogenetic tree using the neighbor-joining approach.

## Electronic supplementary material

Lineage-specific SNPs for genotyping of Mycobacterium tuberculosis clinical isolates
