# Supplementary material for: Lineage-specific SNPs for genotyping of Mycobacterium tuberculosis clinical isolates
Source: Sci Rep. 2017 May 3;7:1425. doi: 10.1038/s41598-017-01580-z (PMC5431204; doi:10.1038/s41598-017-01580-z)

Lineage-specific SNPs for genotyping of *Mycobacterium tuberculosis* clinical isolates

Horng-Yunn Dou[4a#], Chien-Hsing Lin[1a], Yih-Yuan Chen[6], Shiu-Ju Yang[4], Jia-Ru Chang[4], Keh-Ming Wu[1,2,], Ying-Tsong Chen[1,3], Pei-Ju Chin[1,2], Yen-Ming Liu[1], Ih-Jen Su[4], Shih-Feng Tsai[1,2,5#]

[1]Institute of Molecular and Genomic Medicine, National Health Research Institutes, Zhunan, Miaoli, Taiwan
[2]Genome Research Center, National Yang-Ming University, Taipei, Taiwan
[3]Institute of Bioinformatics, National Chung Hsing University, Taichung, Taiwan
[4]Institute of Infectious Diseases and Vaccinology, National Health Research Institutes, Zhunan, Miaoli, Taiwan
[5]Institute of Biomedical Informatics, National Yang-Ming University, Taipei, Taiwan
[6]Department of Biochemical Science and Technology, National Chiayi University, Chia-Yi, Taiwan

[a] Co-first authors

[#] Corresponding authors
Horng-Yunn Dou, PhD
Institute of Infectious Diseases and Vaccinology, National Health Research Institutes, Zhunan, Miaoli County 350, Taiwan
Tel:+886-37-246 166, Extension: 35529
Fax:+886-37-586457
E-mail: hydou@nhri.org.tw

Shih-Feng Tsai, MD, PhD
Division of Molecular and Genomic Medicine, National Health Research Institutes, Zhunan, Miaoli County 350, Taiwan
Fax: +886-37-586459
Tel: +886-37-246166, Extension: 35310
E-mail: petsai@nhri.org.tw

Supplementary Tables

Supplementary Table S1. Genome sequencing and mapping results of the six MTB strains

	by 454 GS-20			by 454 GS-FLX		
**Strain**	**W6**	**M3**	**M7**	**A27**	**A18**	**M24**
Genogroup	modern-Beijing	Haarlem	LAM	T1	EAI	ancient-Beijing
totalReads	899,895	1,031,636	633,450	402,374	308,213	539,007
totalBases	82,327,348	102,358,252	61,414,854	89,801,813	69,425,047	124,997,399
averageLength	91	99	97	223	225	232
**depth (X)$**	**18.66**	**23.20**	**13.92**	**20.36**	**15.74**	**28.33**
mappedReads	846,966	1,008,774	605,942	398,267	304,958	534,763
mappedBases	78,871,442	100,531,219	59,595,662	88,888,588	68,313,195	123,615,730
mappedRate (%)	95.80%	98.22%	97.04%	98.98%	98.40%	98.89%
**totalContigs**	**214**	**267**	**305**	**290**	**290**	**299**
totalContigLength	4,301,167	4,312,708	4,305,621	4,286,613	4,277,247	4,256,888
coverage (%)	98.19%	98.09%	97.98%	97.56%	97.40%	97.43%
**largeContigs***	**134**	**147**	**158**	**200**	**196**	**196**
largeContigLength	4,271,545	4,271,928	4,259,176	4,254,158	4,242,400	4,224,733
avgContigSize	31,877	29,061	26,957	21,271	21,645	21,555
N50ContigSize	64,025	43,790	52,882	37,667	37,913	39,642
largestContig	161,689	132,141	151,052	151,199	101,590	129,955
Q40Bases (%)	99.75%	99.95%	99.59%	99.54%	99.48%	99.68%

$depth = totalBases / length of H37Rv genome
* largeContig: contig length >= 1000 bp
avgContigSize & N50ContigSize are calculated from largeContigs

Mapped by 454 gsMapper Release: 2.3

Supplementary Table S2. High-confidence SNPs with total depth ≥3 and variation rate ≥80% for each site of each strain

Comparing to the reference	**# of SNPs**	**M3**	**W6**	**M7**	**A18**	**A27**	**M24**
Differences in 1 strain	2,376	133	270	317	1,260	136	260
Differences in 2 strains	538	205	325	2	9	206	329
Differences in 3 strains	232	3	228	4	229	3	229
Differences in 4 strains	19	6	19	16	14	3	18
Differences in 5 strains	13	13	12	8	12	9	11
Differences in 6 strains	404	404	404	404	404	404	404
Sum	3,582	764	1,258	751	1,928	761	1,251

Supplementary Table S3. Numbers of strain-specific SNPs used in the genotyping panel in the Sequenom MassArray analysis

**Gene family**	**Substitution**	Isolate (lineage)						**Sum**
		M24 (ancient Beijing)	W6 (modern Beijing)	A18 (EAI)	M7 (LAM)	M3 (Haarlem)	A27 (T)	
PE/PPE	synonymous	5	1	5	4	3	2	60
	non-synonymous	10	6	16	3	3	2	
non-PE/PPE	non-synonymous	10	10	10	10	10	10	60
	**Sum**	25	17	31	17	16	14	120

Supplementary Table S4. SNP discovery in Haarlem and T subtypes of the Euro-American lineage

Data source	Isolate	Lineage	Sublineage	Total # of SNPs	PE/PPE gene family		non-PE/PPE gene family		Intergenic SNPs
					synonymous SNPs	non-synonymous	synonymous SNPs	non-synonymous	
HiSeq2000	A005	*Haarlem*	h3(st227)	2261	126	206	646	1053	230
	D065	*Haarlem*	h3(st742)	984	76	75	308	438	87
	A073	*Haarlem*	h3(st742)	1651	107	154	460	744	186
	B042	*Haarlem*	st36	1645	106	153	449	753	184
	C015	*Haarlem*	h3(st50)	1579	114	113	451	719	182
	W70	*Haarlem*	h3(st50)	1009	65	71	351	435	87
	A074	*T*	T1(st102)	1615	127	148	450	721	169
	B010	*T*	T2-T3(st73)	1638	106	154	457	739	182
	B029	*T*	T2 (st52)	1590	117	160	426	730	157
	W21	*T*	T1,st53	896	69	81	257	397	92
	KVGH295	*T*	T1 like	1039	59	91	318	460	111
	KVGH458	*T*	T3 like	958	78	84	286	421	89
454	M3	*Haarlem*	h3(st742)	813	41	49	276	399	48
	A27	*T*	T1,st53	865	40	41	277	405	102

Supplementary Figures

Supplementary Figure S1. High genetic diversity within the Euro-American lineage. (A) Phylogenetic analysis of Euro-American strains using 4,419 whole-genome SNP markers. The phylogenetic tree was constructed based on Nei's distance using the PHYLIP software (neighbor-joining approach). (B) Principal component analysis (PCA) of Euro-American strains. The genotype data of 4,419 whole-genome SNPs was transformed into numeric values, and then the PCA method was applied to analyze these 14 clinical Euro-American isolates and the H37Rv reference strain using SAS program.

(A)

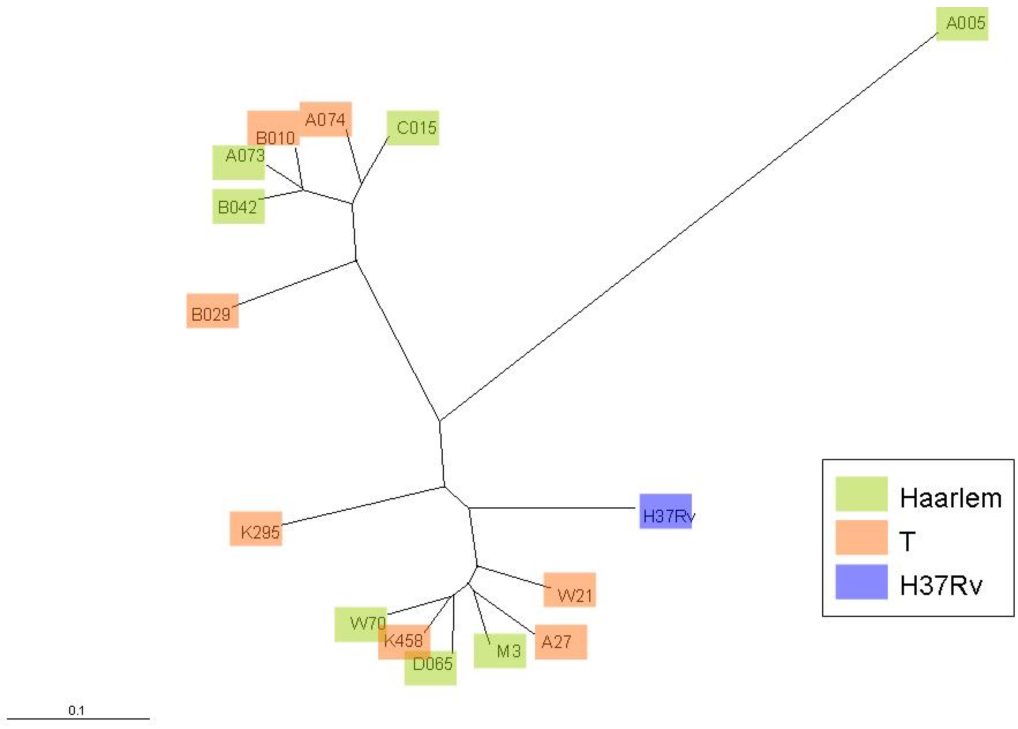

(B)

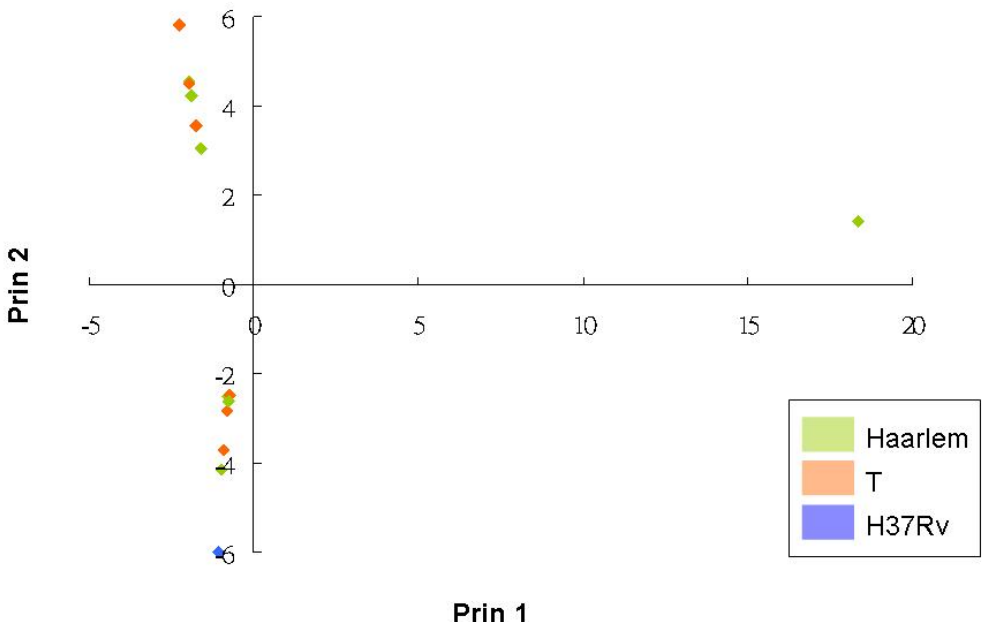

Prin 2
Prin 1
6
4
2
0
-2
-4
-6
-5
0
5
10
15
20
Haarlem
T
H37Rv

Supplementary Figure S2. A minimum spanning tree based on 24-MIRU-VNTR genotyping of 156 *Mycobacterium tuberculosis* isolates. The circles represent different types classified by 24-MIRU-VNTR genotyping and were color-coded according to their spoligotype classification. The sizes of the circles represent the number of isolates sharing the same genotype. (The star symbols indicate strain misclassification by spoligotyping.)

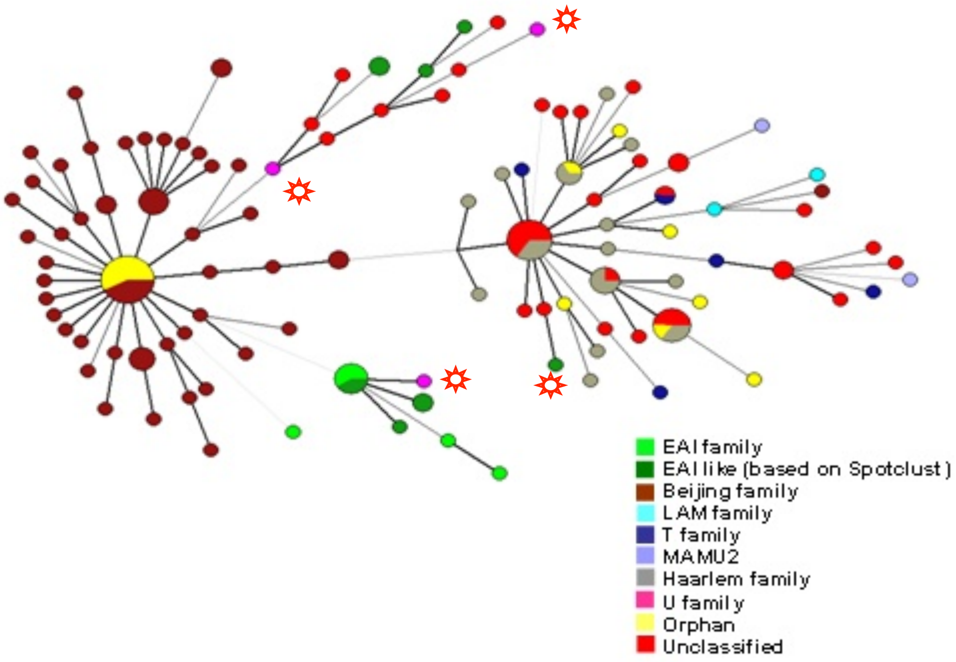

Supplementary Figure S3. New hypothetic subtype definition of the Euro-American lineage. Phylogenetic analysis of Euro-American strains using 4,419 whole-genome SNP markers. The phylogenetic tree was constructed based on Nei's distance using the PHYLIP software (neighbor-joining approach).

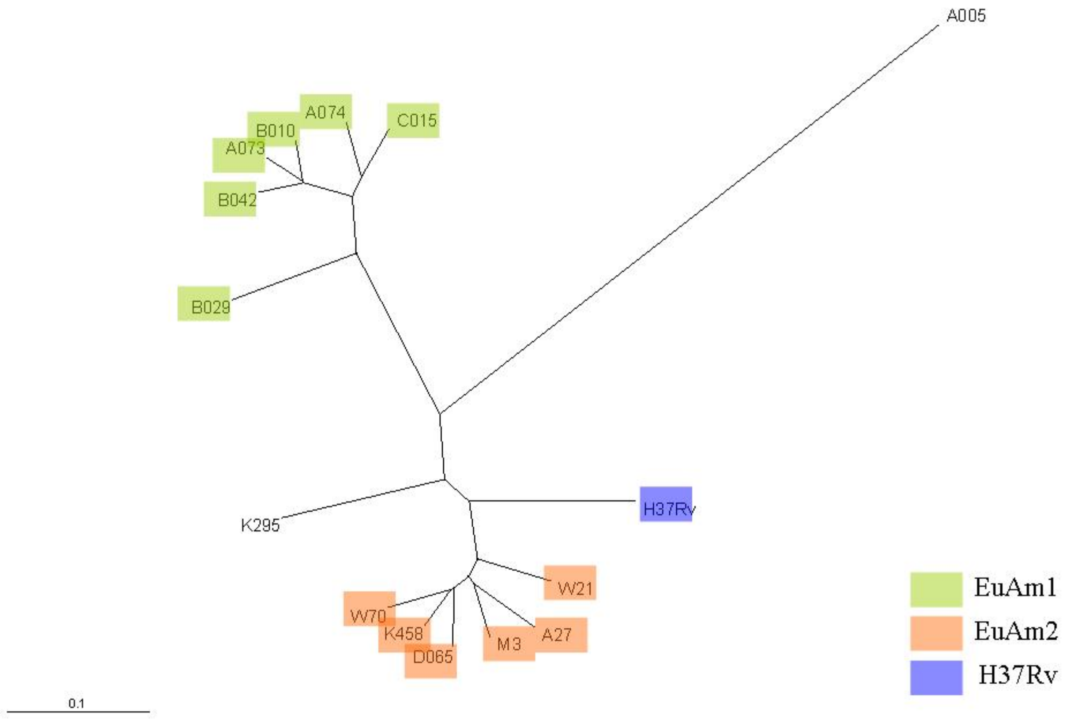

Supplement: Supplementary file 1 — Lineage-specific SNPs for genotyping of Mycobacterium tuberculosis clinical isolates [file 41598_2017_1580_MOESM1_ESM.pdf]
